# Case Report: Heart aneurysm of unknown origin in a two-year-old child diagnosed in the course of Multisystem Inflammatory Syndrome in Children

**DOI:** 10.3389/fcvm.2024.1327996

**Published:** 2024-03-13

**Authors:** Dominika Mystkowska, Michal Galeczka, Wojciech Tarala, Pawel Banaszak, Joanna Sliwka, Szymon Pawlak, Roland Fiszer

**Affiliations:** ^1^ Department of Pediatric Cardiology and Congenital Heart Defects, Faculty of Medical Sciences in Zabrze, Medical University of Silesia, Silesian Center for Heart Diseases, Zabrze, Poland; ^2^ Department of Pediatrics and Pediatric Gastroenterology, College of Medical Sciences, University of Rzeszow, Rzeszow, Poland; ^3^ Department of Cardiac, Vascular and Endovascular Surgery and Transplantology, Faculty of Medical Sciences in Zabrze, Medical University of Silesia, Silesian Center for Heart Diseases, Zabrze, Poland

**Keywords:** SARS-CoV-2 virus, COVID-19, Multisystem Inflammatory Syndrome in Children, heart aneurysm, cardiac surgery

## Abstract

We present a case of a 22-month-old boy with a hypokinetic and thin-walled aneurysm of the left ventricle apex. The lesion was diagnosed during routine echocardiography examination in the course of MIS-C, and its occurrence due to MIS-C is plausible. Cardiac magnetic resonance imaging revealed an akinetic aneurysm of the LV apex with a full-wall ischemic scar. Aortography confirmed a normal course of coronary arteries, with adequate perfusion of essential branches and no evidence of stenosis or aneurysms. The boy underwent consultation with the heart team and was deemed eligible for surgery. The aneurysm was excised up to the margin of healthy tissues, and both the surgery and the periprocedural period were uneventful. Determining the origin of the aneurysm is challenging. The most probable etiology appears to be a congenital lesion. Another consideration is an ischemic lesion that may have resulted from impaired coronary circulation during the complicated course of MIS-C. It is possible that this disturbance resolved spontaneously before aortography was performed. Additionally, a complication of pericarditis cannot be entirely ruled out.

## Introduction

Left ventricular aneurysms are extremely rare findings in the pediatric population (1). The aneurysms can have multiple etiologies, including congenital and acquired ones. Multisystem Inflammatory Syndrome in Children (MIS-C) is a rare condition associated with SARS-CoV-2. Despite the short prevalence period of MIS-C, numerous cardiac complications have already been documented in the literature (2). We report a case of left ventricular aneurysm in a two-year-old child diagnosed during the course of MIS-C, although the relationship between the appearance of the lesion and MIS-C remains uncertain.

## Case report

A 22-month-old boy was admitted to pediatric department due to recurrent fever up to 40 Celsius degrees lasting for 10 days with temporary reaction to antipyretic drugs, with upper respiratory tract infection, and bilateral conjunctivitis. The general state was average, physically rhinitis, redness of the throat, augmented and painful cervical lymph nodes, silent heart murmurs and vesicular murmur over the lung fields, with no heart failure symptoms. Laboratory tests revealed severe anemia (7.7 mg/dl of hemoglobin; *N*: 11.5–14.5), elevated values of C-reactive protein (179.2 mg/L; *N* < 5), NT pro-BNP (3,237 pg/ml; *N* < 125), CK-MB (51.8 U/L; *N* < 24) and slightly elevated troponin I (55.4 pg/ml; *N* < 45). A SARS-CoV-2 IgG antibodies were positive (523 BAU/ml; positive > 33.8), IgM antibodies were negative. ECG was normal, echocardiography showed fluid accumulation in the pericardial sac up to 15.8 mm behind the posterior left ventricle wall, good ejection fraction and non-dilated normal coronary arteries. Empiric antibiotic therapy with cefotaxime and amikacin was started, patient needed blood transfusion, inotropic agents were unnecessary. Colchicine with steroids were introduced to treat suspected pericarditis. Blood tests normalized apart from the elevated NT pro-BNP value (1,717 pg/ml), pericardial effusion reduced (7 mm of fluid behind the left ventricle) and the boy was discharged after 11 days in a good condition on colchicine only.

During a check-up echocardiography performed by the same physician and in the same projections three days after the discharge hypokinetic and thin-walled aneurysm of the left ventricle apex was noticed with dimensions of 20 mm × 16 mm. Contractility was normal and the amount of pericardial fluid was physiological. During subsequent appointments within a month the aneurysm enlarged to 27 mm × 25 mm. The boy remained asymptomatic.

**Figure 1:**
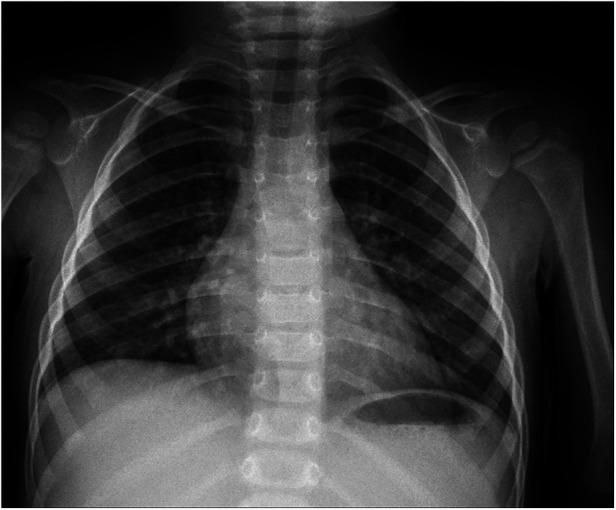
Chest x-ray.

**Figure 2:**
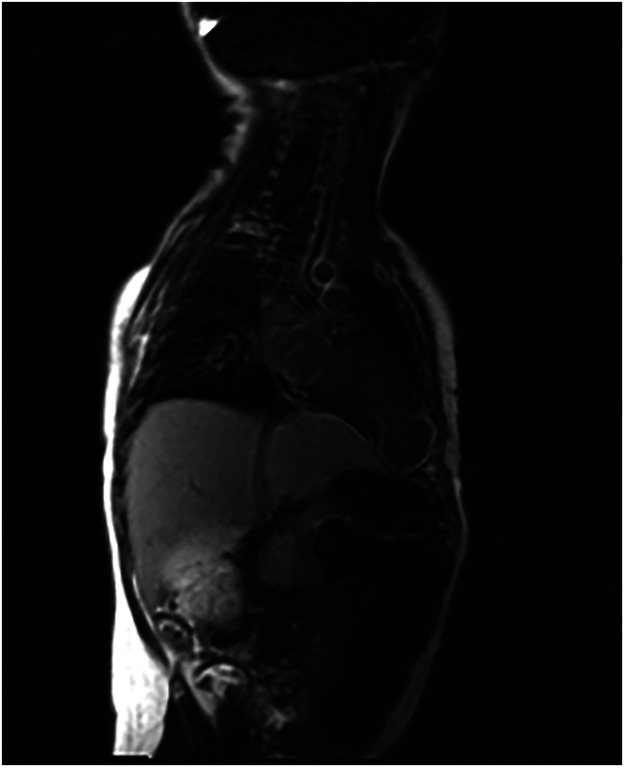
Late gadolinium enhancement over the left ventricle apex aneurysm.

Patient was referred to the tertiary pediatric cardiology center. ECG revealed abnormal negative T waves in V1-V6, 24-h ECG monitoring did not prove any arrhythmias. Laboratory tests pointed out slightly elevated NT pro-BNP value (794 pg/ml), normal value of C-reactive protein, CK-MB and troponins. Transthoracic echocardiography visualized akinetic left ventricle apex aneurysm with wide communication (Figure 1, Supplementary Video S1 and S2). Heart's magnetic resonance imaging (MRI) revealed an additional spherical space originating from the LV apex with thin 2–4 mm walls and filled with blood, and with dimensions of 33 mm × 27 mm (Supplementary Video S3). Late gadolinium enhancement (LGE) indicated full-wall enhancement (up to 100%) of the whole lesion's wall (Figure 2). The signs of edema of the left ventricle's myocardium and slightly reduced ejective fraction (47%) were also diagnosed. Therefore, diagnosis of akinetic aneurysm of the LV apex with the full-wall ischemic scar was established. Diagnostic catheterization with aortography was performed, and proved normal coronary arteries course, with essential branches' sufficient perfusion and with no stenosis or aneurysms. No vasculature of the aforementioned structure was noticed (Supplementary Video S4 and S5). Enoxaparin was started. The boy was consulted on Heart Team and qualified for surgery. In extracorporeal circulation the heart apex aneurysm was cut open, the aneurysm was cut out up to the margin of the healthy tissues and the apex was closed with the continuous double suture (Supplementary Video S6). Both the surgery and the periprocedural period were uneventful. Histopathology examination revealed a fibrous scar with adhering muscle fibers with lysis of myocytes. The 6-month follow-up was uneventful, the boy remains asymptomatic.

## Discussion

A heart aneurysm is exceptionally rare in the pediatric population (3). Typically, aneurysms are classified into congenital and acquired types. Over 800 cases of congenital aneurysms have been documented in the literature (3). Acquired aneurysms can develop due to various factors such as injury (commonly blunt trauma from car accidents), myocardial infarction, myocarditis, pericarditis, or cardiac surgery (4, 5). Congenital left ventricular aneurysms share similarities with acquired aneurysms, often featuring a wide connection to the left ventricle and impaired contractility, as observed in our case (3). These congenital aneurysms are typically located in diaphragmatic, posterobasal, and submitral regions, with approximately 27.5% found at the apex (3). They predominantly consist of fibrous tissue, resembling the composition of the aneurysm in our patient (3, 6). Additionally, congenital heart aneurysms frequently coincide with abnormalities in the coronary arteries, although such abnormalities were not detected in the presented case (6). Congenital heart aneurysms are often asymptomatic, leading to late diagnosis (on average, at 31.5 years). A left ventricle aneurysm may result in congestive heart failure due to volume overload, rupture of the ventricle at the site of the lesion, and ventricular arrhythmic events (3). There are no specific guidelines for management of congenital aneurysms, however, surgical resection should always be considered (17).

A left ventricular diverticulum is an outgrowth emerging from the left ventricular wall, typically at the apex, and contracts in synchrony with the corresponding ventricle, often containing all three layers of the myocardium. However, in the presented case, histopathological examination revealed only fibrous tissue, and the lesion was akinetic (3, 6, 7).

A pseudoaneurysm typically lacks all three layers of cardiac tissue, possesses a narrow neck, and may demonstrate paradoxical (dyskinetic) movement during systole. However, such characteristics were not observed in our patient (4).

The etiology of the presented left ventricular aneurysm is difficult to determine definitively. It is plausible that the lesion could have originated as a congenital aneurysm that went undetected during earlier evaluations. It is possible that the aneurysm was not visualized in standard echocardiographic projections, potentially due to the presence of a significant amount of fluid in the pericardium, which hindered the examination. The observed increase in the size of the aneurysm leads us to speculate that this congenital anomaly might have expanded secondary to inflammation in the pericardium/myocardium during the course of MIS-C.

On the contrary, the presented aneurysm could have been acquired as a result of ischemia or myocarditis during the course of MIS-C. Both MRI and histopathological examinations suggest an ischemic etiology as the primary cause.

The patient met the criteria of MIS-C by CDC during hospitalization in the pediatric department (Supplementary Table S1) (8). The changes in the coronary arteries that arise during MIS-C most often resolve spontaneously within 30 days (9). Aortography was made only in the 3th month of observation, so it makes the hypothesis that our patient had changes in the coronary arteries which caused a myocardial infarction possible. The changes could have resolved spontaneously before aortography was performed. Moreover, aortography does not reveal any changes in coronary microcirculation as well as the most peripheral coronary arteries' segments. Elevated troponins, NT pro-BNP and CK-MB during the acute phase of illness can support this hypothesis. A big amount of fluid in the pericardial sac may have resulted from MIS-C, because in this condition an exudate in a pericardium is a common finding (9). Anemia, conjunctivitis and positive PCT are other not-pathognomonic but rather characteristic findings in MIS-C (10).

Studies show that in 80% of cases of MIS-C the heart might be involved (11). Abnormalities in the coronary arteries are noticed in 14%–48% of patients with MIS-C and are more often described in the cases of male patients with conjunctivitis (8, 11). The boy did not have intravenous immunoglobulin (IVIG) infusion which significantly diminishes the incidence of coronary arteries aneurysms in Kawasaki disease which is a quite similar condition. IVIG should be administrated in MIS-C (10).

The hypothesis that the aneurysm developed due to pericarditis is less probable but also cannot be excluded. Cases of pericarditis complicated by the appearance of a left ventricle aneurysm have been already described (5, 12, 13). In most of the described cases pericarditis was caused by *Staphylococcus aureus*, isolated not only from the pericardial sac, but also from skin or joints (5). Pericardiocentesis was not performed in the presented case, so we cannot prove whether this hypothetical infection had either viral or bacterial origin. The child did not have typical symptoms of *staphylococcal* infection.

In conclusion, heart aneurysms are exceedingly rare in the pediatric population. While a congenital etiology appears to be the most probable explanation for the presented aneurysm, other factors such as ischemia during the course of MIS-C cannot be entirely ruled out.

## Data Availability

The original contributions presented in the study are included in the article/Supplementary Material, further inquiries can be directed to the corresponding author.
