# Supplementary material for: Case Report: Heart aneurysm of unknown origin in a two-year-old child diagnosed in the course of Multisystem Inflammatory Syndrome in Children
Source: Front Cardiovasc Med. 2024 Mar 13;11:1327996. doi: 10.3389/fcvm.2024.1327996 (PMC10965554; doi:10.3389/fcvm.2024.1327996)
Supplement: Supplementary file 7 [file Table1.docx]

**Supplementary table**

| **Case Definitions of Multisystem Inflammatory Syndrome in Children (MIS-C) by the Centers for Disease Control and Prevention (CDC) [8]** | **Signs, symptoms and laboratory tests of our patient during hospitalizations in paediatric departments** |
| --- | --- |
| All 4 findings and age < 21 years old | 20 months old on the onset of illness |
| 1. Fever ≥ 38ºC or subjective ≥ 24h | Recurrent fever up to 40ºC within 1,5 week |
| 2. Laboratory signs of inflammation (positive C-reactive protein, erythrocyte sedimentation rate, fibrinogen, D-dimer, ferritin, LDH, interleukin-6, neutrophilia, and hypoalbuminemia) | C-reactive protein up to 179.2 mg/l (N: <5.0)  fibrinogen: 5.68 g/l (N: 1.64-4.79)  D-dimer: 1751 ng/ml (N <500)  LDH: 350 U/l (N: 195-349)  albumins: 35.4 g/l (N: 38-54)  neutrophils 7.04 x 10^3/ul  (N: 1.5-8)  ferritin: 269.2 ng/ml (N: 30–400)  erythrocyte sedimentation rate and Il-6 – not verified |
| 3. Severe illness requiring hospitalization. | The patient required hospitalization. |
| 4. ≥ 2 organ systems involved (cardiac, renal, respiratory, hematologic, gastrointestinal, dermatologic, and neurologic) | Cardiac: fluid in the pericardial sac, positive troponins, increase of NT pro-BNP up to 3237 pg/ml, CK-MB up to 51.8 U/l  Respiratory: discharge in the nasal passages, redness of the throat, augmented, painful superficial cervical lymph nodes  Gastrointestinal: enlarged liver, slightly enlarged spleen  Haematologic: anaemia (Hb 7.7 g/dl), coagulopathy (prolonged PT: 16.9 s, increased D-dimer) |
| 5. No other possible diagnosis | Negative nose, throat and anus smears, negative virological tests of faeces, negative blood culture and no other abnormalities suggesting another possible diagnosis |
| 6. SARS-CoV-2 infection or exposure, defined as: |  |
| - 1. Positive PCR, serology, or antigen test | Positive serology: IgG 523 BAU/ml, positive if > 33.8) |
| - 2. COVID-19 exposure within 4 weeks before onset | Boy’s mother had an infection described as Covid-19 by a general practitioner one month earlier |
